# Direct antivirals and cognitive impairment in hepatitis C: a clinical-neurophysiologic study

**DOI:** 10.1007/s13365-020-00904-6

**Published:** 2020-09-10

**Authors:** Gloria Vaghi, Benedetta Gori, Gionata Strigaro, Michela Burlone, Rosalba Minisini, Matteo N. Barbaglia, Elena Brigatti, Claudia Varrasi, Mario Pirisi, Roberto Cantello

**Affiliations:** 1 Internal Medicine Department of Translational Medicine, University of Piemonte Orientale, Novara, Italy; 2 Neurology Unit Department of Translational Medicine, University of Piemonte Orientale, Novara, Italy

**Keywords:** Hepatitis C, Cognitive impairment, Neuropsychology, P300 wave, Direct antiviral agents

## Abstract

Cognition was assessed in hepatitis C virus (HCV) patients, who did not meet the criteria for a minimal hepatic encephalopathy. Their liver function was compensated. We then disentangled potential cognitive changes associated with a sustained virologic response at 12 weeks (SVR-12), following treatment with direct antiviral agents (DAAs). We studied 23 selected HCV patients with a battery of standard neuropsychological tests, and with recordings of the P300 wave, a cerebral potential of “cognitive” significance. There was a baseline evaluation (T0) and a second one 6 months later (T1). We had 2 control groups of comparable age and sex, i.e., 15 patients suffering from non-alcoholic fatty liver disease (NAFLD) and 15 healthy subjects. At T0, we detected a significant (*p* < 0.05) cognitive impairment in the HCV group, which involved episodic and working memory, attention, visuospatial and verbal abilities, executive functions, and logic reasoning. The P300 latency was significantly (*p* < 0.05) delayed in the group. At T1, we observed some significant (*p* < 0.05) HCV recovery in given test domains, e.g., memory, executive functions, and reasoning. Accordingly, the P300 latency shortened significantly (*p* < 0.05). HCV patients exhibited subtle cognitive defects, somehow independent of their liver condition, possibly linked to direct or indirect brain involvement by the virus. These defects partly recovered following the SVR-12, as achieved through DAAs. The P300 wave was a valid neurophysiologic counterpart of these changes. DAAs can have a role in the early preservation of cognition in HCVs.

## Introduction

Hepatic encephalopathy (HE) and its mildest expression (minimal HE) are a neuropsychiatric syndrome related to liver failure, with a wide range of severity (Wijdicks 2016). However, hepatitis C virus (HCV) patients not even meeting the definition(s) of minimal HE (Weissenborn 2015) can show a subtle cognitive impairment. This picture, also termed HCV-associated neurocognitive disorder (HCV-AND) affects for instance attention, executive functions, learning/visuospatial abilities, and verbal recall. Its features are indeed different from minimal HE (Monaco et al. 2015). HCV infection can also imply fatigue and depression, and an overall reduced quality of life, which however have no clear relation to cognitive changes (Yeoh et al. 2018).

In the interferon era, several studies after HCV eradication showed improvement not only in quality of life or mood, but also in reaction time (Thein et al. 2007), verbal memory and visuospatial ability (Byrnes et al. 2012), and working memory (Kraus et al. 2013). However, sometimes there were biases such as HIV co-infection, substance abuse, or the neuropsychiatric side effects of peginterferon itself. Some authors questioned any actual improvement, and the whole topic is somehow controversial (Dirks et al. 2017).

Direct antiviral agents (DAAs) can lead to a sustained viral clearance in up to 96.8% of patients, as measured by the absence of HCV RNA sequences in the serum. They lack certain typical side effects of interferon, e.g., fatigue, (Pecoraro et al. 2019). So far, it is not firmly established if DAAs can relieve the HCV-AND syndrome. Some authors described a favorable outcome (Kleefeld et al. 2018), while others did not, or even envisaged some DAA neurotoxicity (Volpato et al. 2017).

We were thus prompted to (a) explore cognition in a selected HCV population not meeting the definition of minimal HE and (b) disentangle any possible cognitive change associated with a successful DAA cycle. To this aim, we combined a battery of standard neuropsychological tests to a recording of cerebral event-related potentials (i.e. the P300 wave). P300 is an “endogenous” brain wave associated with perceptual and cognitive activity. Its amplitude reflects the neural resources involved in a specific cognitive process, while its latency reflects the time course of that process, with a millisecond precision. Among various applications, P300 was thus proposed as a sensitive cognitive index in HCV patients (Kramer et al. 2002). Though not formally indicated for clinical use (Amodio and Montagnese 2015), it may “provide an insight into the cognitive processes in research settings” (Guerit et al. 2009). Recently, its usefulness was strongly revived (Fath-Elbab et al. 2018). Therefore, we hypothesized that P300 would be quite appropriate an instrumental measure, to strengthen the neuropsychological findings expected before and after the DAA treatment.

## Methods

### Subjects

We recruited consecutive patients with HCV infection referred to the Hepatology Clinic of the University Department of Internal Medicine, “Maggiore della Carità” University Hospital, Novara, Italy, between March and November 2017. Inclusion criteria were (a) age > 18 years-old and (b) DAA treatment (a combination of agents from 3 drug classes: protease inhibitors, inhibitors of NS5A complex, and inhibitors of the RNA-dependent RNA polymerase; Appendix). Exclusion criteria were (a) drug abuse; (b) excess alcohol consumption, i.e., > 2UA/day for men and > 1UA/day for women between 18 and 65 years of age; > 1UA/day, above 65 years of age; (c) Child-Pugh-Turcotte score ≥ A7 (Child and Turcotte 1964); (d) central nervous system—CNS—or psychiatric symptoms/signs, particularly depression; (e) fatigue as a consistent complaint; (f) medications acting on the CNS; and (g) hearing loss. Initially, 62 patients met these criteria. However, 6 were rejected for language barriers; 28 did not consent to the study, and 5 were lost at follow-up. Twenty-three HCV patients thus completed the study (10 women, median age 66 years).

As control groups, we had 15 patients with NAFLD (6 women, median age 63 years), whose liver condition was meant to be comparable to the HCV group. We also had 15 HS (7 women, median age 60 years). Demography of controls was similar to HCVs. NAFLD diagnosis was made according to the following criteria: (a) finding of steatosis on liver biopsy, and/or of hyperechoic parenchyma on ultrasounds; (b) exclusion of excess alcohol consumption, as defined previously; (c) exclusion of alternative explanations for steatosis; and (d) exclusion of coexisting causes of chronic liver disease (Chalasani et al. 2018). Moreover, we excluded patients with neurologic/psychiatric symptoms/signs, particularly depression, and with fatigue as a predominant complain.

All subjects, whose IQ was in the normal range, gave their written informed consent to the study. Experiments were approved by the Ethics Committee of the “Maggiore della Carità” University Hospital (# RQ07117), and were performed in accordance with the Declaration of Helsinki.

In principle, all subjects were examined twice, i.e., at baseline (time 0, T0) and 6 months later, i.e., when the 12-week SVR (Chen et al. 2013) of HCV patients had been properly documented (time 1, T1).

### Neuropsychology

Assessment was conducted by a skilled neuropsychologist, blinded to the subject condition. An extensive test battery was used, which lasted approximately 70 min. Global cognition was assessed by means of the Mini Mental State Examination (MMSE) (Folstein et al. 1975) and the Psychometric Hepatic Encephalopathy Score (PHES). PHES is a standardized battery (5 subtests) created to assess neuropsychological functions in patients with chronic hepatitis (Weissenborn et al. 2001). Additionally, verbal learning and episodic memory were tested using the Rey Auditory Verbal Learning Test (RAVLT, immediate and delayed) (Rey 1964). Episodic memory was also assessed by the Digit Forward Span, whereas the Digit Backward Span tested working memory (Wechsler 1958). The Rey-Osterrieth Complex Figure Test (ROCF) was used to evaluate visuospatial abilities/planning in the immediate trial, and episodic and working memory in the 10-min delayed recall trial (Osterrieth 1944). Additional investigation of lexical abilities and executive functions was carried out with the Phonemic (Borkowski et al. 1967) and the Semantic Verbal Fluency tests (Marra et al. 2007). Finally, the verbal judgment test was used to assess logic thinking (Spinnler and Tognoni 1987).

### Neurophysiology: Auditory P300

Studies were carried out between 2:00 and 6:30 p.m. at a standard temperature of 22 °C. Subjects lay comfortably in a dimly illuminated (= ca 30 Lux), partially soundproof room, with eyes open. Overall, we followed the guidelines reported by Duncan et al. (Duncan et al. 2009). The EEG signal was recorded by a BE Light digital machine connected to a multimodal stimulator (EB Neuro Corp., Florence, Italy). Ag/AgCl surface electrodes were attached to the scalp with adhesive electrolyte gel. Active sites were Fz, Cz, Pz, C4, and C3 (10/20 International System), which were referred to averaged mastoids (M1-M2). Electro-oculogram (EOG) was also recorded by electrodes placed at the bilateral outer cantus. Impedance was maintained below 5 kΩ. The filter bandpass was 0.05–100 Hz. Artifacts were rejected automatically (i.e., when the EEG and the EOG amplitude exceeded 100 μV) via software. The sweep was 1000 ms, with a prestimulus analysis of 150 ms. Randomly intermixed standard (80%, 1000 Hz) and target (20%, 2000 Hz) tones were delivered binaurally by headphones at 70 dB SPL. The auditory stimuli lasted 100 ms (including 10 ms rise and fall times), while the inter-stimulus interval (ISI) varied randomly from 1500 to 2000 ms. The first stimulus sequence was presented without instructions (“passive” odd-ball). Later, subjects were instructed to keep a mental record of the target tones and to report their number by the end of the run (“active” odd-ball). Forty-eight artifact-free EEG epochs following the target tones were finally averaged for both paradigms. For all averages, we then identified the P300 wave as the largest positive deflection between 250 and 600 ms after the stimulus onset. We also identified the N100 and N200 components in the time-windows between 60 and 150 and 150–300 ms, respectively. By computer cursors, a neurophysiologist (blinded to subject condition) measured the N100, N200, and P300 latency, and the P300 amplitude as appreciated peak-to-peak (N200-P300), relative to the prestimulus baseline (−150 to 0 ms).

### Data analysis

The statistical analysis was conducted with the SPSS software, ver. 25 (IBM Corp., USA). Almost all biometric/biochemical variables did not show a normal distribution (Shapiro-Wilk test). After grouping, they were thus expressed as median values (25th–75th percentile). Differences among groups were then analyzed by Mann-Whitney *U* tests, Wilcoxon signed-rank tests, or Kruskal-Wallis tests where appropriate. Categorical variables were expressed as frequencies (% of total), and their associations were explored by the Pearson chi-square or Fisher exact tests, where appropriate. The neuropsychological and neurophysiological variables were expressed as mean values ± standard error of the mean (SEM). Baseline (T0) values were compared among groups by means of one-way ANOVA. Comparisons between the T0 and T1 determinations were analyzed by means of repeated-measure ANOVA models. Where necessary, further ANOVAs and post-hoc Tukey HSD tests for multiple comparisons were used to detect differences among subgroups. Significance was set at *p* < 0.05. Correlations between the variables measured were studied by means of Spearman’s rho or Pearson’s *r* coefficients where appropriate.

## Results

### Subject features

No significant difference was detected among the groups for age, sex, and schooling. Considering patients with liver disease, the BMI and the albumin level were significantly (and blood glucose nearly significantly) higher in the NAFLD than in the HCV subgroup, while the reverse was true for AST and ALT measures (Table 1, also for *p* values). The proportion of cirrhotic patients was ca 1/5 (HCV group) and 1/3 (NAFLD group), which implied no significant difference. As far as HCV patients are considered, Table 2 shows the results of blood tests at T0 and T1. There was a significant (*p* < 0.001) improvement of some indexes of liver damage, i.e., AST and ALT, included a reduction in gamma-glutamyl transpeptidase (GGT) (*p* < 0.002).

**Table 1:** Main demographic, clinical, and biochemical features of the participants. Categorical variables are number (%), continuous variables are median values (25–75 percentile)

	Group			
Variable	HCV (*n* = 23)	NAFLD (*n* = 15)	HS (n = 15)	p*
Age, years	66.0 (54.5–72.5)	63.0 (57.0–70.5)	60.0 (56.5–71.0)	0.921
Women, *n* (%)	10 (43)	6 (40)	7 (47)	0.934
Schooling, years	10.0 (7.50–12.0)	9.0 (8.0–11.5)	13.0 (8.00–13.0)	0.287
Albumin, g/L	4.10 (3.99–4.30)	4.40 (4.32–4.55)		0.036
ALP, U/L	158 (129–194)	165 (126–200)		0.950
ALT, U/L	70.5 (51.3–101)	37 (28–76)		0.030
AST, U/L	55 (34–79)	29 (23–37)		0.008
Bilirubin, mg/dl	0.71 (0.65–0.85)	0.75 (0.60–0.91)		0.987
GGT, U/L	39 (28–74)	56 (39–122)		0.162
Glucose, mg/dL	87 (85–93)	97 (91–118)		0.051
PLT, /×10^3^uL	187 (152–225)	209 (188–219)		0.485
BMI, kg/m^2^	24.8 (21.7–27.2)	27.3 (26.00–31.8)		0.042
Cirrhosis, *n* (%)	5 (22)	5 (33)		0.476
HCV_RNA, UI/ml (×10^3^)	474 (214–1614)			
HCV genotype, *n* (%)				
HCV-1a	2 (0.9)			
HCV-1b	17 (74)			
HCV-2	7 (30)			
HCV-3	1 (0.4)			
HCV-4	0 (0)			
Liver stiffness, kPa	6.1 (4.511.4)	8.8 (6.0–14.0)		0.260

*ALT*, alanine amino transferase; *ALP*, alkaline phosphatase; *AST*, aspartate amino tsransferase; *BMI*, body mass index; *GGT*, gamma glutamyl-transpeptidase; *HCV*, hepatitis C virus patients; *HS*, healthy subjects; *NAFLD*, non-alcoholic fatty liver disease patients; *PLT*, platelets. *see methods, for the statistics used

**Table 2:** Biochemical data in patients with chronic hepatitis C at baseline (T0) and 6 months later, i.e., at the time of the 12-week sustained viral response (T1). Variables are median values (25th–75th percentile)

HCV			
Variable	T0	T1	p*
Albumin, g/dL	4.1 (4.0–4.3)	4.2 (4.0–4.6)	0.458
ALP, U/L	158 (129–194)	161 (140–167)	0.849
ALT, U/L	70.5 (51.3–101)	19 (14.8–25.3)	< 0.001
AST, U/L	55 (34–79)	21 (20–32.5)	< 0.001
Bilirubin, mg/dl	0.71 (0.65–0.85)	0.70 (0.58–1.0)	0.584
GGT, U/L	39 (28–74)	19 (15–36)	0.002
PLT, ×10^3/^uL	187 (152–225)	207 (178–248)	0.250

For abbreviations see Table 1. *see Methods, for the statistics used

### Neuropsychology

#### T0

In the HCV patient group, the mean scores on the MMSE, RAVLT, Digit Backward Span, Rey Figure Copy and verbal judgment tests were significantly worse than among HS. Then, if contrasted to the NAFLD group, HCV patients performed significantly worse on the PHES, the RAVLT, and the verbal judgment test, but also on the Rey Complex Figure Delayed and the Semantic Verbal Fluency tests. Interestingly, no significant difference emerged between NAFLDs and HS (Table 3, also for *p* values).

**Table 3:** Baseline (T0) psychometric assessment in the 3 groups (HCV, NAFLD, and HS). Test order is as administered. Data are mean ± SEM. Groups were compared with a one-way ANOVA and between-group comparisons used Tukey HSD post-hoc contrasts

	Group			ANOVA		Post-hoc comparisons (*p*)		
Test	HCV (0)	NAFLD (1)	HS (2)	F	*p*	0 vs 1	0 vs 2	1 vs 2
MMSE	28.4 ± 0.29	29.2 ± 0.30	29.6 ± 0.36	3.96	0.02	–	0.028	–
PHES	− 1.48 ± 0.58	0.53 ± 0.44	1.00 ± 0.45	6.74	0.003	0.027	Not done	Not done
RAVLT	36.1 ± 1.31	42.2 ± 1.66	44.1 ± 1.75	8.05	0.001	0.019	0.002	–
RAVLT delayed	7.91 ± 0.43	8.17 ± 0.57	8.65 ± 0.68	0.48	0.62	–	–	–
Digit Forward Span	6.26 ± 0.31	6.73 ± 0.27	7.13 ± 0.24	2.36	0.105	–	–	–
Digit Backward Span	3.70 ± 0.28	4.33 ± 0.44	5.07 ± 0.32	4.24	0.020	–	0.016	–
Rey Complex Figure Copy	32.5 ± 0.70	34.6 ± 0.50	34.9 ± 0.68	3.97	0.025	–	0.044	–
Rey Complex Figure Delayed	14.1 ± 0.83	18.3 ± 1.47	18.9 ± 1.27	4.54	0.015	0.033	–	–
Phonemic verbal fluency	34.2 ± 2.16	36.8 ± 2.64	42.6 ± 3.24	2.67	0.08	–	–	–
Semantic verbal fluency	17.5 ± 0.86	22.6 ± 1.38	21.5 ± 1.65	5.25	0.008	0.013	–	–
Verbal judgment test	40.8 ± 2.92	50.4 ± 1.67	53.4 ± 0.95	8.30	0.001	0.018	0.001	–

*MMSE* Mini Mental State Examination, *PHES* Psychometric Hepatic Encephalopathy Score, *RAVLT* Rey Auditory Verbal Learning Test

#### T1

A preliminary three-way repeated-measure ANOVA examined the effect of the factors GROUP (HCV, NAFLD, HS), TIME (T0, T1), and TEST (each of the neuropsychological tests). This disclosed a significant GROUP*TIME*TEST within-subject interaction (*F* = 2.391, *p* < 0.001). Thus, the average change (T1-T0) in the test scores was subject to a one-way ANOVA with post-hoc Tukey HSD comparisons among groups. This disclosed a significant improvement of the RAVLT and the verbal fluency scores (semantic and phonemic) in the HCV as compared with the NAFLD group. As to the verbal judgment test, its scores improved among HCVs in a manner that was close to statistical significance in comparison to NAFLDs, and significant in comparison to the HS subgroup (Table 4, also for *p* values).

**Table 4:** Changes in the neuropsychological tests between T1 and T0. After an omnibus repeated-measure ANOVA (see Results), average T1-T0 scores were subject to a one-way ANOVA, and between-group comparisons used post-hoc Tukey HSD contrasts

Test	Score: T1-TO change			ANOVA		Post-hoc comparisons (*p*)		
	HCV (0)	NAFLD (1)	HC (2)	*F*	*p*	0 vs 1	0 vs 2	1 vs 2
MMSE	+ 0.08 (± 0.37)	+ 0.35 (± 0.22)	+ 0.33 (± 0.21)	1.01	0.37	–	–	–
PHES	+ 1.97 (± 1.03)	+ 1.25 (± 1.19)	+ 2.70 (± 1.16)	2.15	0.13	–	–	–
RAVLT	+ 8.00 (± 1.32)	+ 2.81 (± 1.48)	+ 4.22 (± 1.32)	4.04	0.02	0.030	–	–
RAVLT delayed	+ 1.55 (± 0.40)	+ 0.87 (± 0.48)	+ 1.60 (± 0.74)	0.54	0.59	–	–	–
Digit Forward Span	+ 0.22 (± 0.19)	+ 0.07 (± 0.21)	− 0.13 (± 0.22)	0.76	0.47	–	–	–
Digit Backward Span	+ 0.52 (± 0.29)	+ 0.33 (± 0.27)	− 0.13 (± 0.26)	1.39	0.25	–	–	–
Rey Complex Figure Copy	+ 0.89 (± 0.91)	+ 0.43 (± 0.60)	− 1.42 (± 0.69)	2.11	0.13	–	–	–
Rey Complex Figure Delayed	+ 1.98 (± 1.03)	+ 1.25 (± 1.19)	+ 2.70 (± 1.16)	0.35	0.70	–	–	–
Phonemic verbal fluency	+ 5.77 (± 1.69)	− 0.86 (± 2.56)	+ 2.65 (± 1.20)	3.22	0.04	0.044	–	–
Semantic Verbal Fluency	+ 2.93 (±0.91)	− 0.60 (±1.12)	+ 0.80 (±1.13)	3.31	0.04	0.041	–	–
Verbal judgment test	+ 0.22 (± 0.19)	+ 0.07 (± 0.21)	− 0.13 (± 0.22)	5.17	0.01	0.058	0.016	–

Abbreviations: see Table 3

### Neurophysiology: auditory P300

On a preliminary analysis, the latency of the “active” P300 was the sole variable to exhibit changes of interest. Thus, we will report solely about such index, as measured over the midline electrodes Cz, Fz, and Pz, where the wave was best represented.

#### T0

**Fig. 1:**
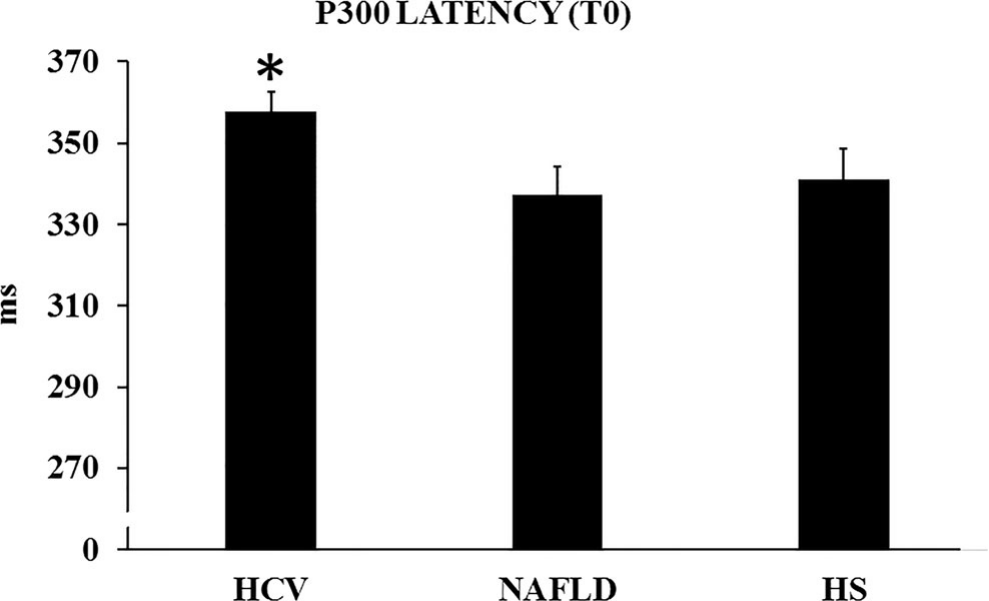
Group values of the P300 latency (average of the Cz, Fz, and Pz electrodes) at T0 in the hepatitis C virus (HCV) and non-alcoholic fatty liver disease (NAFLD) patients, and in healthy subjects (HS). Bars represent standard error of the mean. *p = 0.04 vs NAFLDs

On a repeated-measure ANOVA, the dependent factor was SITE (i.e., Cz, Fz, and Pz), and the independent factor was GROUP. No significant effect emerged for SITE, nor for the SITE*GROUP interaction (*p* > 0.05). Still there was a significant effect of GROUP (*F* = 3.429, *p* < 0.04). On this basis, we averaged for each subject the P300 latency values at all electrodes, which represented the dependent factor in a further one-way ANOVA where GROUP was the independent factor. This disclosed significant results (*F* = 3.432, *p* < 0.04), while post-hoc Tukey HSD tests pointed out a significant difference between HCVs and NAFLDs (*p* = 0.04), while a similar trend was present between HCVs and HS (*p* = 0.1). NAFLDs and HS did not differ significantly (Table 5, Fig. 1). If all controls were grouped (NAFLD+HS), then the difference against HCVs was significant at the *p* = 0.03 level.

**Table 5:** P300 latency measurement in the 3 subject groups. For the baseline determination (T0), separate values for the Cz, Fz, and Pz electrodes are shown, together with their average. Then the change T1-T0 among the groups is shown as the sole average

	Group		
P300 latency (ms)	HCV	NAFLD	HS
Baseline (T0)			
Cz	357.1 ± 5.0	337.5 ± 6.9	341.2 ± 7.6
Fz	358.4 ± 4.6	337.4 ± 6.9	340.7 ± 7.6
Pz	358.2 ± 4.9	337.2 ± 6.9	341.6 ± 7.6
Average 3 sites	357.9 ± 4.8	337.4 ± 6.9	341.2 ± 7.6
Change (T1-T0) Average 3 sites	− 15.3 ± 5.4	− 0.56 ± 0.6	+ 0.52 ± 2.1

Abbreviations: see Table 1

#### T1

**Fig. 2:**
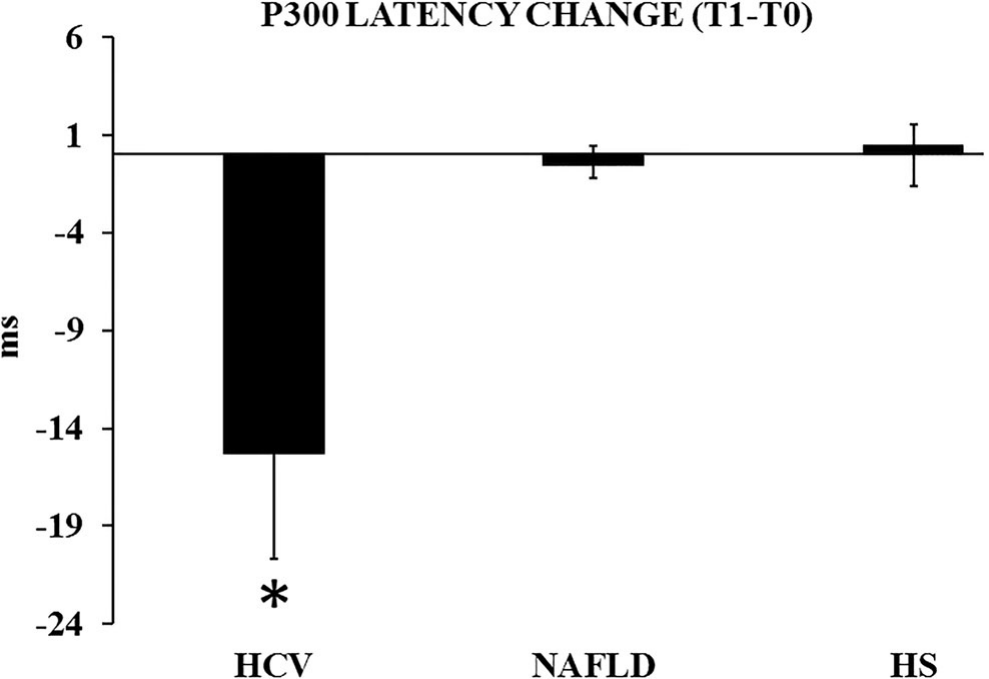
Group values of the change (T1-T0) in the P300 latency (average of the Cz, Fz, and Pz electrodes) across the 3 subject populations. Abbreviations: see Fig. 1. Bars represent standard error of the mean. *p = 0.03 vs NAFLDs and HCVs

**Fig. 3:**
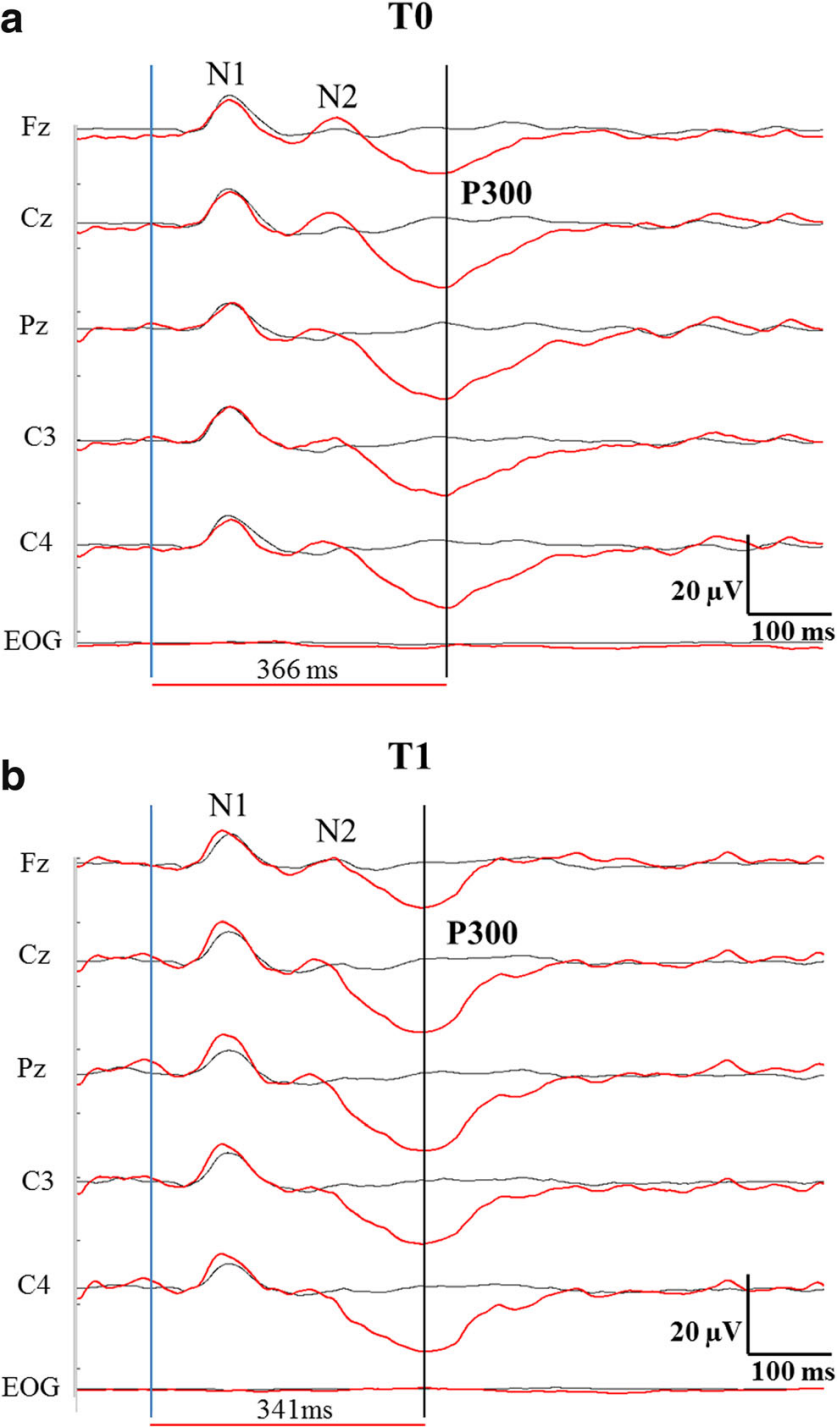
**a**, **b** Typical example of a P300 recording in an HCV patient (#15) prior (**a**) and after (**b**) the treatment with direct antiviral agents (T0 and T1). All electrodes were referred to averaged mastoids. Blue wave: averaged potential evoked by the standard stimulus. Red wave: averaged potential evoked by the oddball stimulus in the “active” paradigm. Blue vertical line: stimulus onset. Black vertical line: P300 latency, which shows a detectable shortening at T1. EOG Electro-oculogram

The P300 latency (average electrodes) at T0 and at T1 was the dependent factor in a repeated measure ANOVA, where the independent factor was GROUP. Here a significant interaction TIME*GROUP emerged (*F* = 3.543, *p* = 0.04). Post-hoc tests revealed that the change T1-T0 distinguished the HCV from the other subgroups significantly (*p* = 0.03), since it exhibited a P300 shortening of about 15 ms, which was lacking in the others (Table 5, Figs. 2 and 3a, b).

#### Correlations

We found no significant correlation between any of the variables studied (*p* > 0.05).

## Discussion

At first, we meant to characterize the HCV-associated neurocognitive disorder (AND) in a highly selected patient sample. To this aim, we compared the HCV psychometry and P300 wave to those of liver patients of equivalent severity, but different etiology (NAFLD), and to normal controls (HS). Selection criteria included, among others, the lack of alcohol or drug consumption, and of depressive symptoms/signs or chronic fatigue. Reportedly, HCV-AND is independent of mood or fatigue (Fortini et al. 2019). Yet, these variables could represent some undue source of bias. Demographic features and IQs were similar across groups, which warranted correct comparisons. Blood test findings were somewhat expected, based on the underlying pathology itself, particularly the AST/ALT enhancement in the HCV as compared with the NAFLD group. Liver function, as expressed by synthesis tests, was relatively preserved in both groups, where the proportion of cirrhotic patients was statistically similar, as was the amount of liver stiffness. Most patients thus suffered from compensated (mild-to-moderate) hepatic disease, with no between-group distinction at the baseline observation (T0). As for T1, we detected a significant lowering of AST/ALT and (to a lesser degree) GGT levels among HCVs. This was somehow expected, due to relieved cytolysis following virologic eradication (SVR-12).

As for neuropsychology at T0, a rather straight-forward finding was the lack of significant differences between NAFLDs and HS. This supports the view that a mild-to-moderate, compensated liver impairment, without HCV infection, would not produce any detectable cognitive defect. By contrast, HCV patients showed several differences in comparison with NAFLD and HS, which sometimes were significant against both, but sometimes against one or the other group. This apparent distinction may simply reflect a lack of statistical power, due to the small sample size, and may not necessarily imply separate interpretations. Anyway, HCVs performed worse than both NAFLDs and HS on a test which explores episodic memory and learning abilities (RAVLT), and on another test, which assesses logic reasoning (verbal judgment). Then, there were differences between the HCV and the HS group on its own, which consisted of a poorer performance on the MMSE, a universal index of global cognition, and on a test which evaluates working memory, such as the Digit Backward Span. The same was true for the ROCF copy, which mainly explores visuospatial abilities, attention, (motor) planning, and working memory. Some tests finally distinguished HCVs from NAFLDs solely, since the former group did worse on the PHES battery, the gold-standard tool to evaluate cognition changes in the liver patient. Meanwhile, it must be stressed that the PHES score at T0 (= − 1.48 in HCVs and = 0.53 in NAFLDs) was far from the cutoff that defines a “minimal” hepatic encephalopathy (= − 4) (Weissenborn 2015). Moreover, HCVs had a poorer performance on the ROCF delayed test, which among others evaluates episodic memory, and on the Semantic Verbal Fluency test, which particularly quantifies the ability to access lexical resources. On the whole then, HCV patients were defective in a series of cognitive domains. This resembles previous reports since the early 2000s (Yarlott et al. 2017). HIV coinfection, alcohol/substance abuse, or high cirrhosis rate might have biased some of these studies (Monaco et al. 2015). However, they provided evidence of impaired verbal memory, attention, and psychomotor speed (Hilsabeck et al. 2002). Also, of defective executive functions (Diamond 2013), encompassing working memory, inhibition, and cognitive flexibility, as well as sustained attention (Lowry et al. 2010). Then, other authors found abnormalities in verbal learning (McAndrews et al. 2005) and verbal recall (Fontana et al. 2005; Lowry et al. 2010). Huckans et al. (Huckans et al. 2009), in a selected sample, reported an impairment of logic reasoning, which tallies the results of ours on the verbal judgment test.

At T0, we found a delayed P300 wave latency in the “active oddball” paradigm, which distinguished HCVs from NAFLDs significantly, and close to significance from HS. If both control groups were averaged, the difference attained a *p* level = 0.03. P300 is an event-related potential, having a nearly simultaneous latency over widespread cerebral regions. It may be generated by many independent sources, or by a “central integrated system” with a large cortical representation. P300 latency correlates with information processing speed (particularly stimulus categorization time), reaction time, and age (Picton 1992). This wave has been proposed as an early neurophysiologic marker of cognitive impairment in several neurological disorders, especially dementia (Duncan et al. 2009). It was also recommended in HCV patients with subclinical cognitive impairment, where it proved independent of depression, fatigue, or changes in the quality of life, and capable to overcome the inherent bias of neuropsychological batteries or the uncertainties of self-rating scales (Kramer et al. 2002). Later studies questioned the P300 practical value in the clinical assessment of hepatic encephalopathy, since routine or quantitative EEG was found superior (Amodio et al. 2005). The International Society for Hepatic Encephalopathy and Nitrogen Metabolism (ISHEN) still preserved some role for the P300 in the research setting (Guerit et al. 2009). Most recently, the P300 validity has been strongly revived by Fath-Elbab et al. (2018), since this index could disclose “minimal and subclinical impairment of cognitive function at early stages of chronic hepatitis with accuracy”. Having controlled for confounding factors such age and alcohol consumption (Duncan et al. 2009), we thus value the P300 latency lengthening in HCVs as an objective counterpart of the subtle cognitive changes observed. Perhaps, supplementary EEG recordings would have been helpful, which is another limit of the present work. Yet, additional instrumental investigations were deemed to hamper the patient compliance.

Severity of liver involvement was equivalent among patients. Thus, the neuropsycho-physiological differences between HCVs and NAFLDs imply a responsibility of the viral infection, and its consequences. Besides, there are many evidences for a neurobiological origin of the HCV-AND, such as changes in the choline/creatinine or N-acetyl-aspartyl-glutamate/creatine ratios, or increased myoinositol, on proton magnetic resonance spectroscopy in various brain regions (Monaco et al. 2015). Some authors even found brain micro-structural abnormalities using diffusion tensor MRI (Thames et al. 2015). HCV infection was associated with glial activation and increased myelin turnover. Viral particles and specific proteins were detected in given brain areas during HIV co-infection, and localized within cells of the astro- or microglial line (Letendre et al. 2007). Often, a neuroinflammatory neural response was envisaged, with a prominent role for cytokines (Yarlott et al. 2017). Moreover, aminergic transmission alterations were shown in HCV-AND patients by means single-photon emission computerized tomography (SPECT) (Weissenborn et al. 2006). Similarly, fluorodeoxyglucose PET studies revealed metabolic abnormalities in several cortical regions (Heeren et al. 2011).

Secondly, we re-evaluated all subjects when HCVs had achieved their SVR-12 (T1). Here we relied on the premise that practice effects represented a systematic error across groups in the test-retest experimental design. On this assumption, we associate the SVR-12 with a significant cognitive improvement, limited to definite domains. This is the case for the functions tested by the RAVLT immediate test, i.e., predominantly short-term auditory-verbal memory, rate of learning, learning strategies, retroactive, and proactive interference. Then, changes in the Verbal Fluency support improvement in the access to lexical resources, as well as in cognitive flexibility and inhibitory control. These, in turn, represent key aspects of the “executive” machinery. Improvement of logic reasoning/thinking, as revealed by the verbal judgment test, would finally be in line with the previous effects. In the first place, such improvement may be related to better liver conditions, as shown by the decreased cytolysis. Other factors are however expected, since we previously showed that a mildly/moderately deranged liver function per se cannot justify cognitive changes. Then, one could invoke an overall improved quality of life, mood, or fatigue, which is somehow expected at the time of the SVR-12 (Juanbeltz et al. 2018). We do not report here these variables, which may represent another limit of the study, yet they were an exclusion criterion. Moreover, the HCV-AND would evolve as a separate entity (Dirks et al. 2017). Thus, a direct or indirect effect of the viral clearance onto the brain must be seriously considered.

The P300 latency showed a significant shortening at T1, which distinguished HCVs from the other subjects. This can reflect, in the first instance, the improved attentional and stimulus-processing abilities, which emerge by the psychometric assessment, as described previously. Particularly, the P300 latency mainly reflects the speed of the cognitive process involved in the experimental task (Duncan et al. 2009). To explain the P300 latency delay at T0, one must invoke the many neurobiological consequences of the HCV infection (detailed above). These may well impair the complex neural systems underlying the P300 physiology. In turn, viral eradication at T1 would consistently relieve such widespread functional impairment, reversing the P300 delay to a significant (*p* = 0.03) extent. From another perspective, the P300 change appears to strengthen, in an objective manner, the data obtained on clinical grounds. Possibly, further clinical-neurophysiologic evaluations at later time-points would have strengthened the findings, which we add to the study limits. Still, the present results somehow resemble those of Kleefeld et al. (2018). In a study of 22 patients, including HIV-coinfected subjects, these authors reported improvement in visual memory/learning, executive functions, verbal fluency, processing speed, and motor skills, on the achievement of SVR-12 after DAAs. Similarly, in the work by Hamdy et al. (2018), the sole Montreal Cognitive Assessment (MOCA) test was used to assess 62 HCV patients, and beneficial effects were found at SVR-12. By contrast, Volpato et al. (2017) compared 10 HCV patients with cirrhosis to 12 post-liver transplant HCVs. At the end of the DAA treatment, some worsening (on psychometry and EEG) emerged in the former, but not the latter group. At 6 months, this effect disappeared. No cognitive improvement by DAAs was however seen.

Unfortunately, we detected no significant correlations between the variables studied (demography, liver conditions, neuropsychology, and neurophysiology). This is possibly due to the lack of statistical power, which also rendered it unfeasible any data stratification, nor an analysis at the individual level.

## Conclusions

Despite the limitations mentioned above, and considering the high patient selection, we confirm that HCV patients exhibit a peculiar type of subtle cognitive impairment (HCV-AND). This is mostly independent of liver derangement, as disclosed by the comparison with NAFLDs. Therefore, it must be ascribed to the direct or indirect effects of the viral infection onto the brain. Viral clearance through DAAs implies a cognitive improvement in given functional domains. The P300 wave can document these changes. DAAs may thus have a role in the early preservation of higher-order neural abilities in HCVs.

## Appendix

In all HCV patients, treatment with direct-acting antiviral agents (DAAs) lasted 12 weeks. The drugs used were elbasvir/grazoprevir 50 mg/100 mg (Zepatier®) (11 patients); sofosbuvir/velpatasvir 400 mg/100 mg (Epclusa®) (8 patients); ombitasvir/paritaprevir/ ritonavir 12.5 mg/75 mg/50 mg + dasabuvir 250 mg (Viekirax® + Exviera®) (3 patients); daclatasvir/sofosbuvir 60 mg (Daklinza ® + sofosbuvir) (1 patient).
